# Supplementary material for: Changing temporal context in human temporal lobe promotes memory of distinct episodes
Source: Nat Commun. 2019 Jan 14;10:203. doi: 10.1038/s41467-018-08189-4 (PMC6331638; doi:10.1038/s41467-018-08189-4)
Supplement: Supplementary file 1 — Supplementary Information [file 41467_2018_8189_MOESM1_ESM.pdf]

Supplementary Information

Changing temporal context in human temporal lobe promotes memory of distinct episodes

El-Kalliny et al.

Supplementary Methods

Behavioral tasks

In the paired associates memory task, we presented lists composed of six pairs of nouns chosen at random and without replacement from a pool of high-frequency nouns. Word pairs were presented sequentially and appeared in capital letters at the center of the screen, with both words of a given pair presented simultaneously with one above the other. Study word pairs were separated from their corresponding recall cue by a minimum lag of two study or test items. During the study period (encoding), the first word pair was preceded by a row of asterisks that appeared on the screen for 275 ± 25 ms, followed by a blank screen of 625 ± 125 ms, before the first word pair appeared. Each word pair remained on the screen for 4000 ms and was followed by a blank interstimulus interval (ISI) of 1000 ms in one version of the task and 750 ± 250 ms in a second version of the task. Immediately after the final word in each list, participants performed a distractor task (20 s) consisting of a series of arithmetic problems of the form A + B + C = ?, where A, B, and C were randomly chosen integers ranging from 1 to 9. After the distractor task, one randomly chosen word from each study pair was shown in succession, and the participant was asked to recall the corresponding word from each pair by vocalizing a response into a microphone. Participants either responded with the correct word, responded with an incorrect word (intrusions), made no response, or vocalized the word 'pass'. Vocalizations were recorded digitally and then scored manually for analysis. Each session consisted of up to 25 lists of this encoding-distractor-recall procedure, such that a single experimental session contained up to 150 total word pairs. Non-stimulation sessions consisted of 24.48 ± 1.42 lists, and stimulation sessions all consisted of 25 lists.

In the free recall memory task, we presented lists of 12 words chosen at random and without replacement from a pool of high-frequency nouns. Words were presented sequentially and appeared in capital letters at the center of the screen. Each word remained on the screen for 1600 ms, followed by a blank inter-stimulus interval (ISI), randomly jittered from 750-1000 ms. Immediately after the final word in each list, participants performed a distractor task (20 s) consisting of a series of arithmetic problems of the form A + B + C = ?, where A, B, and C were randomly chosen integers ranging from 1 to 9. After the distractor task, participants were given 30 s to verbally recall as many words as possible from the list in any order; vocal responses were digitally recorded and later manually scored for analysis. Each session consisted of up to 25 lists of this encoding-distractor-recall procedure, such that a single experimental session contained up to 300 total words. Non-stimulation sessions consisted of 23.05 ± 2.56 lists, and stimulation sessions all consisted of 25 lists.

Multivariate classification

During experimental sessions that did not involve electrical stimulation (record-only sessions), we trained a logistic regression classifier to discriminate encoding-related activity predictive of whether a word was later remembered or forgotten (1, 2). We used as inputs to the regression spectral power averaged across the time dimension for each word encoding epoch (0 - 2,000 ms relative to word onset in the paired associates task; 0 - 1,366 ms relative to word onset in the free recall task). Thus, the features for each individual item were the average power across time, at each

of eight frequencies (logarithmically spaced between 3 and 180 Hz; Morlet wavelets; wave number = 5 cycles), for each implanted electrode across the brain. We used L2-penalization (3) and set the penalty parameter (C) to 2.4104 in free recall and 0.048 in paired associates, based on the optimal penalty parameter calculated across pre-existing datasets of free recall and paired associates subjects (1). In both tasks, successful memory formation is consistently accompanied by a pattern of increases in high-frequency activity and decreases in low-frequency activity during the encoding period (Supplementary Fig. S3) (1, 4). Classification of good memory encoding states in general identify when this pattern emerges in the neural data (1).

Closed-loop electrical stimulation

At the start of each stimulation session, we determined the safe amplitude for stimulation using a mapping procedure in which stimulation was applied at 0.5 mA while a neurologist monitored for afterdischarges. This procedure was repeated, incrementing the amplitude in steps of 0.5 mA, up to a maximum of 1.5 mA for depth contacts and 2.5 mA for cortical surface contacts. These maximum amplitudes were chosen to be below the afterdischarge threshold and below accepted safety limits for charge density (5).

We then applied stimulation on 11 randomly-chosen lists. During each stimulation list, we applied the classifier weights to the pattern of spectral power across all electrodes, computed from 0 - 2,000 ms following each word pair presentation in the paired associates task, and 0 - 1,366 ms following each word presentation in the free recall task. If the classifier generated an output probability below 0.5, corresponding to a lower likelihood of successful encoding, we immediately triggered 500 ms of stimulation. The motivation for this approach was that previous evidence has suggested that stimulation applied during poor memory encoding states can enhance memory performance (1). The classifier outputs were centered around 0.5, designed such that stimulation would be triggered on approximately half of the items in each stimulation list. We delivered stimulation using charge-balanced biphasic rectangular pulses (pulse width 300 μs) at (10, 25, 50, 100, or 200) Hz frequency and (0.25 to 1.50) mA amplitude at depth contacts and (0.25 to 2.50) mA for cortical surface contacts (0.25 mA steps) (2). Stimulation was not applied during the first three lists, and baseline spectral data collected during these lists was used for the z-transform normalization of the input features. During non-stimulation lists, spectral power features and classifier output were computed identically to stimulation lists, but stimulation was disabled. Data captured during non-stimulation lists were used for continued z-transform normalization of input features.

We passed electrical current through a single pair of adjacent electrode contacts. As the electrode locations were determined strictly by the monitoring needs of the clinicians, we used a combination of anatomical and functional information to select stimulation sites. If available, we prioritized electrodes in lateral temporal cortex, in particular the middle portion of the middle temporal gyrus. To choose among electrodes in regions in which more than one electrode was available, we selected the electrode pair demonstrating the largest differences in the high frequency (70 - 200 Hz) power between correct and incorrect trials during the record-only sessions (subsequent memory effect, SME) (2). In cases in which no lateral temporal cortex contacts were available, we selected an electrode pair at or near the largest SME elsewhere in the brain (two participants in paired associates, three participants in free recall). For a

subset of participants, the particular amplitude and frequency used for stimulation were chosen based on a pre-test in which we stimulated the brain at each parameter combination, while the patient was at rest (no experimental task; 3 of 7 participants in paired associates, 7 of 8 participants in free recall). The frequency-amplitude combination that maximized the change in classifier output was used in the closed-loop memory task.

References

[1] Ezzyat, Y. *et al.* Direct Brain Stimulation Modulates Encoding States and Memory Performance in Humans. *Current Biology* **27**, 1251–1258 (2017).

[2] Ezzyat, Y. *et al.* Closed-loop stimulation of temporal cortex rescues functional networks and improves memory. *Nature Communications* **9** (2018).

[3] Hastie, T., Tibshirani, R. & Friedman, J. The Elements of Statistical Learning. *Springer 2001* **18**, 746 (2001).

[4] Greenberg, J. A., Burke, J. F., Haque, R., Kahana, M. J. & Zaghloul, K. A. Decreases in theta and increases in high frequency activity underlie associative memory encoding. *Neuroimage* **114**, 257–263 (2015).

[5] Shannon, R. V. A Model of Safe Levels for Electrical Stimulation. *IEEE Transactions on Biomedical Engineering* **39**, 424–426 (1992).

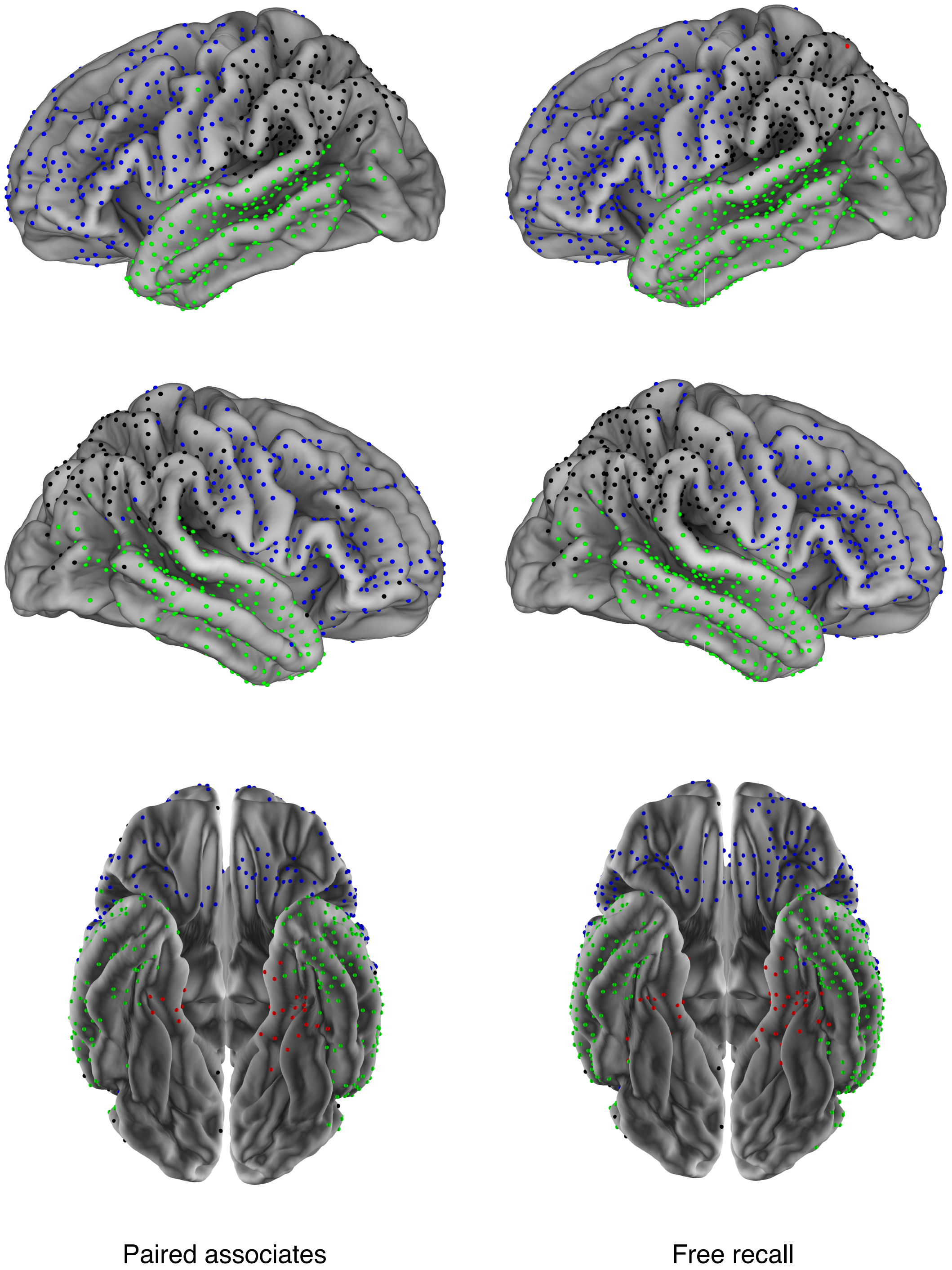

Supplementary Figure 1. Electrode coverage in all participants who performed either the free recall or paired associates task projected on to a standard brain surface. Electrodes are color coded by brain region for subsequent analysis (lateral temporal lobe, green; medial temporal lobe, red; frontal lobe, blue; parietal lobe, black). Depth electrodes not shown.

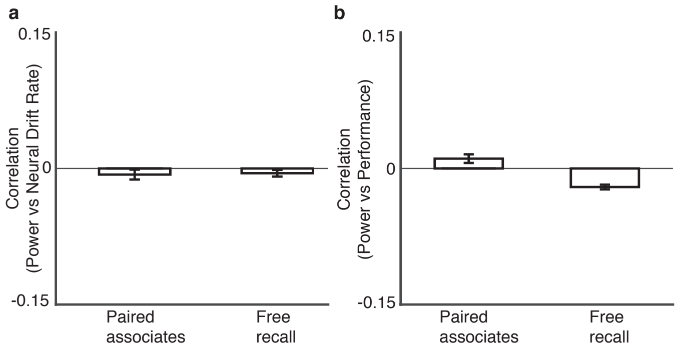

Supplementary Figure 2. Relation between performance and neural drift rate is not related to overall power changes. It is possible that lists that exhibit greater neural drift do so not because of changes in a multivariate pattern of power across different electrodes, but because of univariate changes in power, across all electrodes. We controlled for this possibility by computing the overall rate of change in interstimulus, low frequency power (3-12 Hz), averaged across all electrodes and items in each list. (*A*) There was no relation between rate of change in average oscillatory power and memory performance (paired associates, $t(27) = 0.384$, $p = 0.703$; free recall, $t(47) = -0.917$, $p = 0.362$, one-sample t-test). (*B*) There was no relationship between rate of change in average oscillatory power and rate of neural drift (paired associates, $t(27) = -0.218$, $p = 0.829$, one-sample; free recall, $t(47) = -0.186$, $p = 0.853$, one-sample t-test). Error bars represent SEM across 28 paired associates participants.

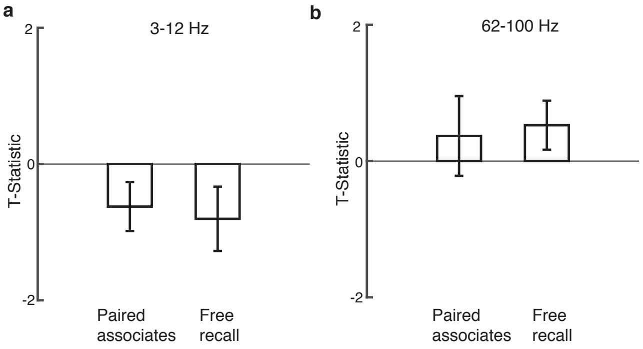

Supplementary Figure 3. To ensure that the paired associates and free recall participants demonstrate similar patterns of neural activity in general, we conducted an analysis to confirm previous reports that decreases in low-frequency power and increases in high-frequency power underlie successful memory formation in both tasks. We examined differences in spectral power during the encoding period (0 to 1.5 s relative to stimulus onset), in both a low-frequency (3-12 Hz) and high-frequency (62-100 Hz) band. For each participant, we calculated the difference in power between successfully retrieved and unsuccessfully retrieved memories independently for each electrode in the temporal lobe, then calculated a t-statistic of power differences across electrodes. We observed that the distributions of t-statistics, across subjects, were comparable between paired associates and free recall participants, both in low-frequency and high-frequency changes. Error bars represent SEM across 28 paired associates participants, and 48 free recall participants.

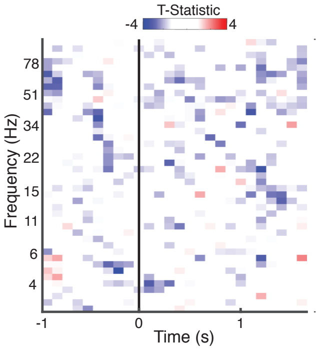

Supplementary Figure 4. Neural drift rate and memory performance in free recall, by frequency and time. As in the paired associates analysis, for each combination of frequency and time, we examined the relation between rate of drift in neural activity and performance on the free recall task. We generated one correlation coefficient for each participant, indicating the relation between rate of drift and performance, across all lists. We then tested whether the distribution of correlation coefficients, at each time-frequency combination, was significantly different than zero, using a nonparametric clustering-based procedure (see Materials and Methods). There were no significant clusters ($p > .05$) at which rate of neural drift was related to memory performance.

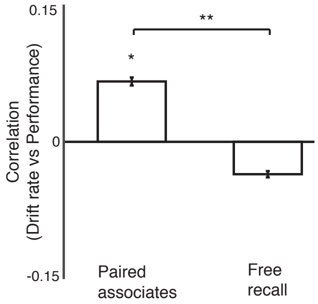

Supplementary Figure 5. We repeated our analysis by defining a single unit of lag in free recall as interstimulus epochs that were separated by two intervening item presentations, to match the elapsed time between word pairs in the paired associates task. We found a similar difference in the relation between drift rate and memory performance (paired associates vs free recall, $t(74) = 2.76$, $p = .0072$, two-sample t-test; free recall, $t(47) = -1.43$, $p = .159$, one-sample t-test), confirming that the difference between paired associates and free recall is not related to the differing length of encoding epochs in the two tasks. Error bars represent SEM across 28 paired associates participants, and 48 free recall participants.

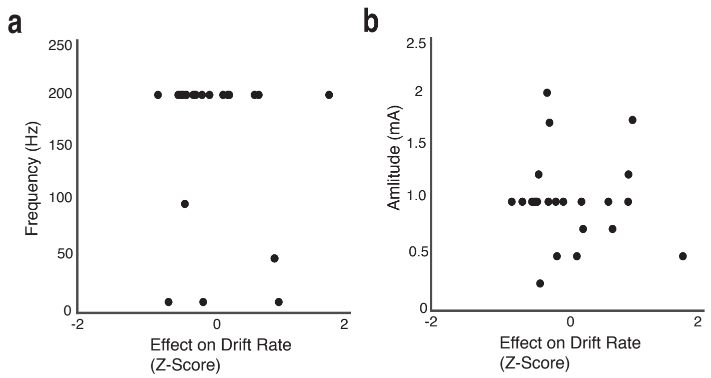

Supplementary Figure 6. We combined data from stimulation sessions across the two tasks, and examined whether sessions in which stimulation caused an increase in drift rate were associated with higher or lower values of pulse frequency or amplitude, compared to sessions in which stimulation caused a decrease in drift rate. We found no significant results when looking at the effect of stimulation across all sessions (pulse frequency, $t(20) = -1.10$, $p = 0.283$; pulse amplitude, $t(20) = -1.15$, $p = 0.265$, one-sample t-test) or when looking at the effect of stimulation applied via electrodes in lateral temporal cortex (pulse frequency, $t(14) = -1.18$, $p = 0.256$; pulse amplitude, $t(14) = -0.138$, $p = 0.892$, one-sample t-test).

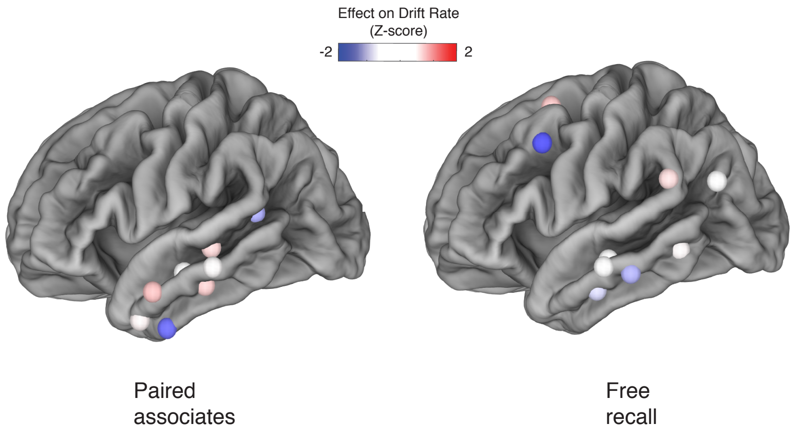

Supplementary Figure 7. Electrodes through which electrical stimulation was applied, for paired associates and free recall participants. Depth electrodes not shown.

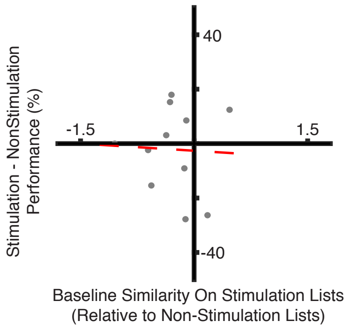

Supplementary Figure 8. There was no significant relation between the effects of stimulation on memory performance and its effects on baseline similarity (paired associates, $rho = 0.100$, $p = 0.762$, permutation test; free recall, $rho = -0.078$, $p = 0.817$, permutation test).

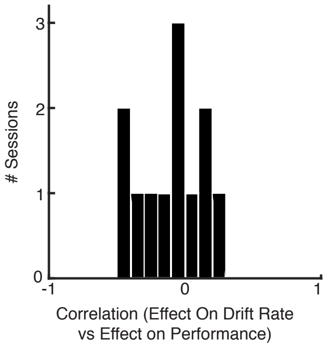

Supplementary Figure 9. For each participant receiving stimulation during the free recall task (N=8, 12 unique stimulation sites), we z-transformed the rate of neural drift in interstimulus 3-12 Hz activity and memory performance on each stimulation list, relative to the distributions of drift rate and performance observed on non-stimulation lists. We correlated the effect of stimulation on performance with the effect of stimulation on drift rate, across the 11 stimulation lists of each free recall session. We did not find a consistent correlation between effect of stimulation on drift rate and effect of stimulation on performance ($r = -.109 \pm 0.067$, $t(11) = -1.63$, $p = 0.132$, one-sample t-test).

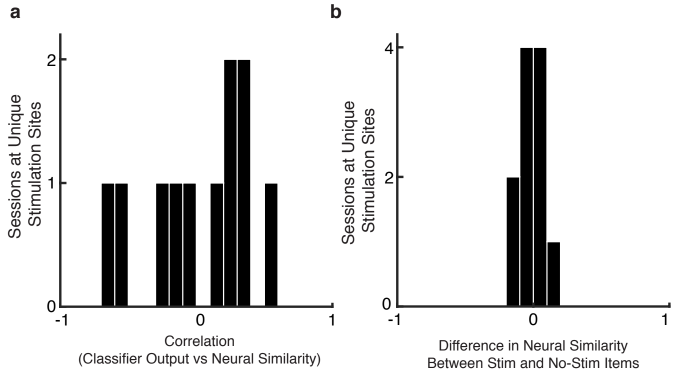

Supplementary Figure 10. During each stimulation list of a stimulation session, the decision of whether to stimulate or not was under the control of a classifier trained on previous data to predict when the brain is in a poor encoding state, and to deliver stimulation only on those trials. The classifier was designed to capture trial-specific activity that is predictive of good encoding, whereas the rate of neural drift describes a between-trial phenomenon. However, there is a possibility that the decision of the classifier to stimulate is linked to the neural drift rate around that point in time. To examine this possibility, we first conducted a list-level analysis, in which we examined the relation between the number of items stimulated during each list (a proxy of average classifier output throughout that list), and the neural drift rate calculated for that list. We generated one correlation coefficient per unique stimulation site, and across participants, found that there was no consistent relationship ($t(10) = 0.284$, $p = 0.782$, one-sample t-test). We then conducted a second analysis in which we examined whether the decision to stimulate was related to the degree of neural similarity at the level of individual items. Given that the metric of neural drift rate cannot be calculated at the level of single items, for each item we measured absolute cosine similarity between the prior two interstimulus intervals. We used this measure as a surrogate metric for drift rate. We separately examined the values of similarity for the items of the stimulation lists in which stimulation was applied, and the items of the stimulation lists in which no stimulation was applied. We calculated the difference between similarity for these two groups, and across participants, found no consistent relationship ($t(10) = -0.945$, $p = 0.367$, two-sample t-test).
