# Supplementary material for: Changing temporal context in human temporal lobe promotes memory of distinct episodes
Source: Nat Commun. 2019 Jan 14;10:203. doi: 10.1038/s41467-018-08189-4 (PMC6331638; doi:10.1038/s41467-018-08189-4)
Supplement: Supplementary file 3 — Reporting Summary [file 41467_2018_8189_MOESM3_ESM.pdf]

nature research

Corresponding author(s): Kareem Zaghloul

# Reporting Summary

Nature Research wishes to improve the reproducibility of the work that we publish. This form provides structure for consistency and transparency in reporting. For further information on Nature Research policies, see Authors & Referees and the Editorial Policy Checklist.

## Statistical parameters

When statistical analyses are reported, confirm that the following items are present in the relevant location (e.g. figure legend, table legend, main text, or Methods section).

| n/a | Confirmed | |
|---|---|---|
| ☐ | ☒ | The exact sample size (*n*) for each experimental group/condition, given as a discrete number and unit of measurement |
| ☐ | ☒ | An indication of whether measurements were taken from distinct samples or whether the same sample was measured repeatedly |
| ☐ | ☒ | The statistical test(s) used AND whether they are one- or two-sided<br>*Only common tests should be described solely by name; describe more complex techniques in the Methods section.* |
| ☐ | ☒ | A description of all covariates tested |
| ☐ | ☒ | A description of any assumptions or corrections, such as tests of normality and adjustment for multiple comparisons |
| ☐ | ☒ | A full description of the statistics including central tendency (e.g. means) or other basic estimates (e.g. regression coefficient) AND variation (e.g. standard deviation) or associated estimates of uncertainty (e.g. confidence intervals) |
| ☐ | ☒ | For null hypothesis testing, the test statistic (e.g. *F*, *t*, *r*) with confidence intervals, effect sizes, degrees of freedom and *P* value noted<br>*Give P values as exact values whenever suitable.* |
| ☒ | ☐ | For Bayesian analysis, information on the choice of priors and Markov chain Monte Carlo settings |
| ☒ | ☐ | For hierarchical and complex designs, identification of the appropriate level for tests and full reporting of outcomes |
| ☐ | ☒ | Estimates of effect sizes (e.g. Cohen's *d*, Pearson's *r*), indicating how they were calculated |
| ☐ | ☒ | Clearly defined error bars<br>*State explicitly what error bars represent (e.g. SD, SE, CI)* |

*Our web collection on statistics for biologists may be useful.*

## Software and code

Policy information about availability of computer code

| | |
|---|---|
| Data collection | Data was collected using various intracranial EEG (iEEG) acquisition systems. |
| Data analysis | Imaging data were analyzed using both FSL Brain Extraction Tool and FLIRT software packages. Custom MATLAB analysis code was used, and is available upon request. |

For manuscripts utilizing custom algorithms or software that are central to the research but not yet described in published literature, software must be made available to editors/reviewers upon request. We strongly encourage code deposition in a community repository (e.g. GitHub). See the Nature Research guidelines for submitting code & software for further information.

## Data

Policy information about availability of data

All manuscripts must include a data availability statement. This statement should provide the following information, where applicable:

- Accession codes, unique identifiers, or web links for publicly available datasets
- A list of figures that have associated raw data
- A description of any restrictions on data availability

Processed data used in this study can be found at: https://neuroscience.nih.gov/ninds/zaghloul/downloads.html.

# Field-specific reporting

Please select the best fit for your research. If you are not sure, read the appropriate sections before making your selection.

☒ Life sciences ☐ Behavioural & social sciences ☐ Ecological, evolutionary & environmental sciences

For a reference copy of the document with all sections, see nature.com/authors/policies/ReportingSummary-flat.pdf

# Life sciences study design

All studies must disclose on these points even when the disclosure is negative.

| | |
|---|---|
| Sample size | In order to aggregate data within a given brain region across participants, we require a minimum of five participants with intracranial electrodes implanted within that brain regions. Due to clinical considerations, each participant has a different arrangement of implanted surface and depth electrodes. Given this variability and based on our previous work, we estimated that we needed at minimum 12 participants to perform a task before we can draw significant conclusions regarding changes in neural activity in any one brain region. No explicit power calculations were performed to determine sample sizes. However, we far exceeded our minimum sample size, and based on electrode coverage (Fig. S1), believe that our data provides more than sufficient coverage over the majority of the cortical surface. |
| Data exclusions | We excluded data from each session in which less than 20 lists were completed. Our analyses involve a comparison of neural and behavioral activity across lists, and thus this criterion ensures that we have sufficient power within each session to examine a correlation. This led to the exclusion of all data from two participants. We also excluded data from participants who had insufficient electrode coverage in the temporal lobe (less than 10 electrodes, following removal of artifactual channels). Our analyses involve defining a vector which describes a spatial pattern of activity in the temporal lobe. This criterion ensures that the pattern of activity across this brain region is well represented. This criterion led to exclusion data from three participants who performed the paired associates task, and five participants who performed the free recall task. |
| Replication | We did not replicate these results in a separate cohort of participants. The data presented here were captured over three years from intracranial recordings in human neurosurgical patients receiving treatment for epilepsy at various hospitals, and are thus extremely rare. |
| Randomization | Randomization of participants was not relevant to this study and participants were therefore not allocated into separate groups. Each participant completed experimental sessions in which analyses were conducted comparing neural activity between task conditions. |
| Blinding | Blinding was not relevant to this study. All participants performed the same behavioral tasks. |

# Reporting for specific materials, systems and methods

## Materials & experimental systems

| n/a | Involved in the study |
|---|---|
| ☒ | ☐ Unique biological materials |
| ☒ | ☐ Antibodies |
| ☒ | ☐ Eukaryotic cell lines |
| ☒ | ☐ Palaeontology |
| ☒ | ☐ Animals and other organisms |
| ☐ | ☒ Human research participants |

## Methods

| n/a | Involved in the study |
|---|---|
| ☒ | ☐ ChIP-seq |
| ☒ | ☐ Flow cytometry |
| ☒ | ☐ MRI-based neuroimaging |

## Human research participants

Policy information about studies involving human research participants

| | |
|---|---|
| Population characteristics | Amongst the 76 participants from whom data was analyzed: 41 male, age 36.2 +/- 1.32 years. |
| Recruitment | Participants were recruited on the basis of clinical need for surgical epilepsy localization. |
